# A systematic review on health systems integrated approach towards elimination of HIV, syphilis and viral hepatitis as public health threats in sub-Saharan Africa

**DOI:** 10.11604/pamj.2026.53.159.49740

**Published:** 2026-04-14

**Authors:** Newten Handireketi, Pisirai Ndarukwa, Tafadzwa Mindu, Moses John Chimbari

**Affiliations:** 1 School of Public Health, University of KwaZulu-Natal, Durban, South Africa,; 2 Department of Health Systems, National Institute of Health Research, Harare, Zimbabwe,; 3 Department of Health Sciences, Bindura University of Science Education, Bindura, Zimbabwe,; 4 Department of Psychiatry and Social Behavioural Science, National University of Science and Technology, Bulawayo, Zimbabwe,; 5 Durban University of Science and Technology, Dean's Office, Faculty of Health Sciences, P.O Box 1334, Durban 4000, South Africa

**Keywords:** HIV, syphilis, hepatitis, integration, sub-Saharan Africa

## Abstract

Health systems (HSs) in sub-Saharan Africa (SSA) face significant challenges. Integrated HSs approach has been considered a feasible option for eliminating HIV, syphilis and hepatitis B (triple elimination). We performed a systematic review to determine the extent of utilisation of the integrated HSs approach towards triple elimination in SSA countries. We registered the systematic review protocol on PROSPERO (ID: CRD420251011221). We adopted Arksey and O'Malley's review methodology framework for article searching on databases: Medline (using PubMed interface), EMBASE and EBSCOHOST. Studies published from March 2015 to March 2025 were assessed by two reviewers independently. Joanna Briggs' checklist was used to assess the quality of included studies. Thematic analysis was performed, and results were reported according to the Enhancing Transparency in Reporting the Synthesis of Qualitative Research (ENTREQ) statement. A total of 533 studies were identified from databases. Eighteen studies were fully reviewed. Five themes were developed: Effectiveness of HSs integration, Barriers to HSs integration, Facilitators for HSs integrated approach on triple elimination, Improved case detection, and Recommendations to HSs integration. All themes but one were developed from sub-themes that were built around six blocks of the health systems, according to the World Health Organization. Progress in the integrated approach exists. A fully integrated HSs approach for triple elimination remains unmet in SSA. Facilitators and barriers within the WHO's HSs building blocks can work for or against an integrated approach towards triple elimination, offering a roadmap for future efforts to strengthen HSs. Further enquiry into HSs integrated approach needs to be considered towards triple elimination.

## Introduction

Mother-to-child (vertical) transmission (MTCT) of HIV, syphilis, and hepatitis B virus (HBV) significantly contributes to morbidity and mortality, especially in low- and middle-income countries (LMICs). It has been targeted for triple elimination through an integrated approach [1]. In 2023, the MTCT rate, including perinatal and postnatal infections among 1.2 million pregnant women living with HIV, was about 10%, against a 2030 target of 5% [2]. Syphilis accounted for about 2 million cases among pregnant women, with around 460 000 new cases of miscarriages and stillbirths. Approximately 270 000 incidences of congenital syphilis, and close to 270 000 cases of low birth weight children when mothers received poor antenatal care (ANC) follow-ups were observed globally [3]. HBV was also reported at about 65 million prevalent cases globally among reproductive-aged women, with 9 out of every 10 infected mothers transmitting the virus to their children perinatally [4]. The global community has committed to the triple elimination of vertical transmission of HIV, syphilis, and HBV as a public health priority that contributes to Sustainable Development Goal (SDG) 3 - "to ensure healthy lives and promote well-being for all at all ages" [5]. However, findings from 18 sub-Saharan African (SSA) countries surveyed showed that 10 of them were tested for only HIV and syphilis at least once during pregnancy [6], resulting in only a minority of HBV-infected individuals being diagnosed and treated. The three diseases have similar characteristics, such as their epidemiology, disease interactions and core interventions for tackling them, thus providing opportunities for an integrated health-systems approach for elimination of their MTCT [7].

The World Health Organization (WHO) proposed an integrated and coordinated approach towards triple elimination, contributing to achieving universal health coverage [8]. The Global Fund to Fight AIDS, tuberculosis, and malaria has supported the new focus of triple elimination of vertical transmission among pregnant and breastfeeding women by encouraging applicants "to prioritise integrated approaches to eliminate mother-to-child transmission of HIV, syphilis, and hepatitis B (triple elimination). This includes screening for HIV, syphilis, and HBV at ANC" [9]. There is no universal definition, shared conceptual understanding or single model of care that can be replicated locally about integrated care, and due to its polymorphous nature, little evidence exists to determine that it works [10]. A health-system-wide (health system) approach is needed to effectively support both transformation and actions towards achieving integrated health service delivery [11]. Yet change at the health system level is mainly dependent on addressing all intersecting concepts. A health system comprises all the organizations, institutions, resources, and people with a primary purpose of improving health [12]. WHO has described the 6 HS building blocks as: i) service delivery, (ii) health workforce, (iii) health information systems, (iv) access to essential medicines, (v) financing, and (vi) leadership/governance [13]. United Nations International Children's Emergency Fund (UNICEF) encourages health systems to deliver integrated packages of service with appropriate quality for all children and women [14]. The Global health sector strategies on HIV, viral hepatitis and sexually transmitted infections, for the period 2022-2030, have identified an integrated health systems approach as a key strategy for addressing the triple burden of HIV, syphilis, and HBV [15]. This integrated approach involves the coordination of services, resources, and interventions for the management of these diseases within a single, cohesive health system framework. By leveraging shared infrastructure, personnel, and healthcare resources, an integrated HSs approach promises to enhance efficiency, reduce fragmentation, and improve outcomes in the fight against these diseases.

Health systems are increasingly becoming integrated in developed countries such as the United States, the United Kingdom and Australia [16]. Despite the theoretical promise of an HSs integrated approach, limited studies have specifically explored its impact, effectiveness and challenges in achieving the triple elimination, particularly in the context of SSA. Relatively little attention has been given to the integration of services targeting HIV, syphilis, and hepatitis within a comprehensive framework, as most studies have focused on smaller subsets of interventions. Furthermore, there is no dominant health system integration theory, model or framework that has been identified, suggesting that a one-size-fits-all solution may not be effective [17]. This gap in the evidence base means that there is insufficient understanding of how integrated health systems can be designed, implemented, and sustained in SSA, where the health system infrastructure may be strained, and resources are often limited.

SSA's health systems face various challenges that include deficiencies in health infrastructure, inadequately trained healthcare professionals, persistent issues of equity in access, high out-of-pocket expenses [18] and low healthcare financing, especially syphilis and HBV programs, which remain grossly underfunded. The need to better understand the challenges and experiences with the integrated HSs approach in this SSA context is paramount to identify both the opportunities and barriers to successful integration. Understanding these challenges could inform policy, guide future health system strengthening efforts, and ensure that interventions are tailored to local needs and capacities. Moreover, identifying best practices and lessons learned from both successes and failures could offer valuable insights to policymakers and health practitioners striving to achieve the ambitious goal of eliminating HIV, syphilis, and HBV as public health threats.

Necessity for measures to enhance syphilis and HBV testing and treatment among pregnant women is highlighted by the fact that, despite significant progress in reducing MTCT of HIV, gaps still exist in preventing MTCT of HBV and syphilis, especially for newborns [19]. A systematic review on the studies reporting the HSs integrated approach towards the elimination of these diseases in SSA is therefore compelling. We carried out a systematic review aimed at identifying and retrieving international evidence on the utilization of an integrated HSs approach on the elimination of HIV, syphilis and viral hepatitis as public health threats in SSA. This study aimed to appraise and synthesize results of the search to inform practice, policy and in some cases, further research. We considered a systematic review due to its ‘gold standard' way of synthesizing findings of several studies that have investigated similar questions [20,21], like HSs integrated approach towards triple elimination. A systematic review is essential to provide a comprehensive synthesis of existing evidence, highlighting key insights on the effectiveness, facilitators, and barriers to full integration of services for triple elimination. By compiling findings across diverse contexts and settings, in this review, we intended to contribute to the body of knowledge necessary to inform evidence-based decision-making towards triple elimination. This helps to guide future interventions aimed at the elimination of the triple burden in SSA through the innovative HSs integrated approach.

## Methods

We developed a protocol using the Enhancing Transparency in Reporting the Synthesis of Qualitative Research (ENTREQ) statement [22] and registered it on the PROSPERO database (ref: CRD420251011221). The registration indicated that no similar work had been done or was currently being done elsewhere.

**Study design:** this systematic review focused on the evidence on impact, effectiveness, facilitators and barriers to an integrated approach towards HIV, syphilis and viral hepatitis elimination as public health threats. A synthesis of qualitative data was performed to generate findings that were meaningful, relevant and appropriate for informing policy and practices on an integrated approach towards triple elimination.

**Study eligibility criteria:** the review included peer-appraised studies from sub-Saharan Africa. Only peer-reviewed articles published in English, reporting an integrated approach to HIV, syphilis and viral hepatitis were included. The studies published from March 2015 up to March 2025 were included.

**Study participants:** the review included all studies that reported on an integrated Health Systems (HSs) approach related to HIV, syphilis and viral hepatitis in sub-Saharan African countries.

**Type of interventions:** studies that focused on various forms of integration in the Health Systems in sub-Saharan Africa were included in the review. The impact, effectiveness, barriers and facilitators for an integrated approach to healthcare services were explored. Alternative synonyms to the concept of the integrated health systems approach were used interchangeably during the search for available articles. Several definitions about integrated care services exist but the following WHO definition was generally used in this review: “Integrated health services delivery is defined as an approach to strengthen people-centred health systems through the promotion of the comprehensive delivery of quality services across the life-course, designed according to the multidimensional needs of the population and the individual and delivered by a coordinated multidisciplinary team of providers working across settings and levels of care”. It should be effectively managed to ensure optimal outcomes and the appropriate use of resources based on the best available evidence, with feedback loops to continuously improve performance and to tackle upstream causes of ill health and to promote well-being through inter-sectoral and multi-sectoral actions" [23].

**Types of outcome measures:** the phenomenon of interest in this review was the elimination of HIV/syphilis/viral hepatitis as a public health threat and the associated synonyms of the concept.

**Study exclusion criteria:** studies not available in English, conference abstracts, books or grey literature and editorial comments were excluded by the reviewers.

**Search strategy:** a combination of Medical Subject Headings (MeSH) combining the Phenomena, Intervention, Comparator, Outcome (PICO) and Region (SSA) was used to identify peer-reviewed articles on integrated services related to HIV, syphilis and hepatitis to determine their impact, effectiveness, barriers and facilitators (Annex 1). The databases searched were PubMed/MEDLINE, Scopus/Embase (Elsevier), Ebsco-Host, CINAHL and Google Scholar. The search term used for the articles on PubMed is in Annex 1.

**Selection of study and process of data management:** the ENTREQ guidelines were used for reporting qualitative systematic reviews. Two reviewers independently screened all titles from the retrieved studies. The eligible studies were imported into Rayyan for abstract, followed by full-text screening after removing duplicates. Data extraction was then done through Elicit/Google Doc/Excel, after which a full write-up was prepared. A third reviewer was tasked to break any discordance(s) between the two reviewers, and a consensus was reached among the three. The full texts of all selected relevant studies meeting the inclusion criteria were used for the final framework synthesis.

**Quality appraisal:** all retrieved articles eligible for inclusion underwent a quality assessment process during the synthesis of results. The quality appraisal was conducted by two independent reviewers using the Joanna Briggs Institute's Critical Appraisal Checklist for Qualitative Research assessment tool [24], used in systematic reviews. In case of discordance between the two reviewers, a third reviewer gave an independent opinion and a discussion with the first two reviewers to resolve the difference(s) was held. After following the process, we were able to assess whether the included studies met the quality assessment criteria during the synthesis of results.

**Data extraction:** data from sub-Saharan African (SSA) eligible studies were independently extracted onto a data extraction form by two review authors and populated with variables pertaining to phenomena of interest, intervention, comparator and outcome. A third review author double-checked and verified the extracted articles. An adapted Joanna Briggs Institute (JBI) data abstraction format was used by the reviewers [25]. The following study characteristics were extracted: name of the first author, year of publication, data collection period, country where the study was conducted, study design, study population, sampling procedures, sample size and data collection procedures, as well as impact/effectiveness/challenge(s)/opportunities of the integrated approach to HIV, syphilis and hepatitis.

**Data synthesis and analysis:** we performed stratified analysis and reporting of findings from clusters to demonstrate differences among specific groups. Such subgroups included patient age groups, health workers and policy-makers in healthcare. Data were analysed and synthesised through a thematic framework analysis approach. The advantages of the approach were that descriptive themes remained close to reported data in primary studies, and by developing analytical themes, thematic synthesis could extend beyond the descriptive themes. This improved our appreciation of the impact, effectiveness, challenges and opportunities of an integrated approach towards triple elimination of HIV, syphilis and viral hepatitis. The five stages of framework synthesis were followed to synthesise qualitative data.

**Familiarisation with the data:** the first reviewer started by familiarising himself with the data, relating it to the review's objectives and identifying recurring patterns in the studies. A pre-determined thematic framework was used by the reviewers to develop emerging themes from studies selected for our analysis. This framework offered a detailed list of likely factors that could contribute to challenges and/or possible strategies used to overcome these challenges in an integrated approach towards triple elimination of HIV, syphilis and viral hepatitis.

**Indexing:** the two reviewers read the extracted information independently to identify themes accordingly, in line with the predetermined thematic framework. They also searched for any additional emergent themes, allowing for revision of the framework as new themes emerged. The review team discussed and reached an agreement on the themes. The process was done on all studies until there were no more new themes emerging. The identified themes were coded based on the identified patterns in the data. Each of the primary studies was indexed using the codes related to the themes from the framework. Parts of the studies were indexed with one or more codes where it was appropriate.

**Charting:** data from the selected studies were sorted by theme and presented in the form of an analysis table by the reviewers. Rows and columns of the analysis table showed the studies and related themes for comparing findings from the different studies.

**Mapping and interpretation:** the reviewers mapped the range and nature of the integrated health systems towards triple elimination phenomena using charts to define the identified concepts. The review determined associations among the themes to clarify our findings in line with the objectives and emerging themes.

**Ethics and dissemination:** ethical approval was not sought by the authors of this systematic review for conducting the study because there was no need for access to confidential and sensitive individual-level data. The study went through PROSPERO registration (Number: CRD420251011221). Results and findings from this systematic review will be made available through open access to a broad range of stakeholders. Policy-makers and funders in HIV, syphilis and hepatitis programming will be engaged to consider the review results for evidence-informed decision-making.

## Results

**Figure 1:**
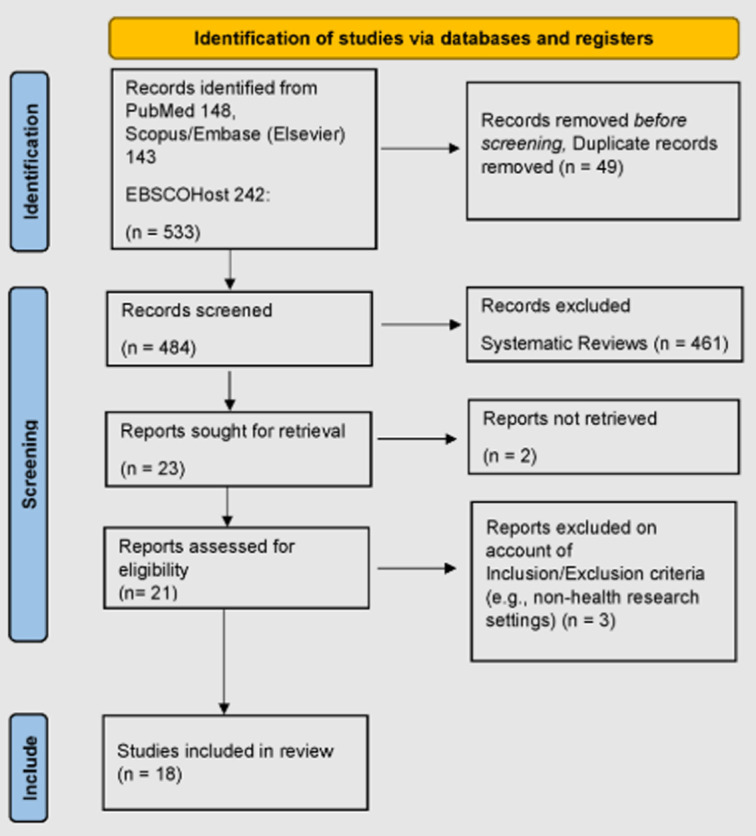
PRISMA diagram for the HSS integrated approach systematic review search

**Results of search and description of included studies:** a total of 533 articles were identified in the initial search through PubMed, Scopus/Embase (Elsevier) and EBSCOHost. After removal of duplicates, 484 articles were subjected to title, abstract and full text screening. Finally, 18 articles were included as shown in the PRISMA flow diagram depicted in Figure 1. The studies included were conducted in sub-Saharan Africa or among countries that included those in the region and published between 2015 and 2025.

**Study quality:** an assessment of the quality of studies that were included was made, and it ranged from 7 to 10 as shown in Table 1. The quality of the included studies, as evaluated using the Joanna Briggs Institute (JBI) critical appraisal tools were of moderate to high methodological quality. Seven of the studies achieved the maximum score of 10, reflecting strong adherence to key methodological criteria. In this systematic review, no studies among those that were assessed for quality were excluded based on quality, as all met the minimum threshold for inclusion.

**Table 1:** quality assessment of studies reporting an integrated approach to HIV, syphilis and HBV management in sub-Saharan Africa

Study	JBI's critical appraisal questions												
	Sample size	Q1	Q2	Q3	Q4	Q5	Q6	Q7	Q8	Q9	Q10	Score	Overall Appraisal
Mathebula *et al*. 2020	1822	Y	Y	Y	Y	Y	N	Y	Y	Y	Y	9	Include
Thompson *et al*. 2021	90	U	Y	Y	Y	Y	Y	Y	Y	Y	Y	9	Include
Alege *et al*. 2025	30	Y	Y	N	Y	Y	Y	Y	Y	Y	Y	10	Include
Toskin *et al*. 2020	Not specified	U	Y	Y	Y	Y	U	Y	U	Y	Y	7	Include
Mullick *et al*. 2023	22505	N	Y	Y	Y	Y	Y	Y	Y	Y	Y	9	Include
Cohn *et al*. 2021	Not specified	Y	Y	Y	Y	Y	N	Y	Y	Y	Y	9	Include
Newman *et al*. 2015	Not specified	Y	Y	Y	Y	Y	Y	U	Y	Y	Y	9	Include
Phili *et al*. 2015	10806	Y	Y	Y	Y	Y	Y	Y	Y	Y	Y	10	Include
Ciccacci *et al*. 2024	1051	N	Y	Y	Y	Y	Y	Y	Y	Y	Y	10	Include
Young *et al*. 2019	18	Y	Y	Y	Y	Y	Y	Y	Y	Y	Y	10	Include
Ejalu *et al*. 2021	3121	Y	Y	Y	Y	Y	Y	U	Y	Y	Y	9	Include
Young *et al*. 2019	183	U	Y	Y	Y	Y	N	Y	Y	Y	Y	10	Include
Manabe *et al*. 2015	584	Y	Y	Y	Y	Y	Y	Y	Y	Y	Y	10	Include
Baseke *et al*. 2015	89	U	Y	Y	Y	Y	U	Y	Y	Y	Y	9	Include
Peeling *et al*. 2017	Not specified	U	Y	Y	Y	Y	N	Y	Y	Y	Y	9	Include
Tucker *et al*. 2017	27	Y	Y	Y	Y	Y	U	Y	Y	Y	Y	9	Include
Balira *et al*. 2015	76	Y	Y	Y	Y	Y	Y	Y	Y	Y	Y	10	Include
Hutin *et al*. 2017	Not specified	U	Y	Y	Y	Y	N	U	Y	Y	Y	8	Include

**Types of included studies:** overall, the included articles examined data from cross-sectional feasibility studies, exploratory qualitative descriptive and longitudinal field intervention studies. The details of the studies that were included are shown in Table 2, Table 2.1, Table 2.2, Table 2.3 and Table 2.4. A total of 18 studies reporting an integrated approach to HIV, syphilis, and hepatitis B management in sub-Saharan Africa were summarised in Table 2, Table 2.1, Table 2.2, Table 2.3 and Table 2.4. The majority (13) of the studies were cross-sectional, while there were 3 longitudinal studies, 1 feasibility study, and 1 exploratory study. The findings from studies that were included and analysed to identify patterns using thematic analysis are shown in Table 3.

**Table 2:** summary of findings for studies reporting an integrated approach to HIV, syphilis and hepatitis B management in sub-Saharan Africa

Author, Publication Year	Country	Study title	Study aim	The study methodology	Sample characteristics	Summary of Findings
Mathebula, *et al*. 2020	South Africa	Factors associated with repeat genital symptoms among sexually transmitted infection service attendees in South Africa, 2015 - 2016	To describe the prevalence of and factors associated with repeat genital symptoms among STI service attendees at primary care facilities.	A cross-sectional study was carried out among 7 STI primary care facilities. Participants (1822) provided demographic, clinical information and genital specimens. Multivariable logistic regression was done.	Seven STI primary care facilities from 5 provinces in SA participated in this study.	There was a high prevalence of repeat genital symptoms ‒ many without STI aetiology. Identified factors highlight the need for improved integration of HIV and STI prevention and management.
Thompson *et al*. 2021	DRC	Arresting vertical transmission of hepatitis B virus (AVERT-HBV) in pregnant women and their neonates in the DRC: a feasibility study	To evaluate the feasibility and acceptability of adding HBV testing and treatment of pregnant women, as well as the birth-dose vaccination of HBV-exposed infants to the HIV PMTCT programme infrastructure in the DRC.	A feasibility study was conducted in two maternity centres in Kinshasa. Pregnant women were screened for HBV under the HIV PMTCT programme. The positives were included in the study. Those with viral load ≥200 000 IU/mL or HBeAg positivity, or both, were considered as having HBV of high MTCT risk and initiated on oral TDF up to 12 weeks post-partum. HBV-exposed infants received a birth dose of HBV within 24 h. All women were followed up for 24 weeks.	A total of 90 participants from two maternity centres in Kinshasa (Binza and Kingasani) were recruited.	Adding HBV screening and treatment of pregnant women and infant birth-dose vaccination to existing HIV PMTCT platforms is feasible. HBV birth-dose vaccination, integrated within the Expanded Programme on Immunisation and HIV PMTCT programme, could accelerate progress toward HBV elimination in Africa.
Alege *et al*. 2025	Uganda	Barriers and facilitators of integrated HBV, HCV, and HIV screening among pregnant mothers and Newborns attending maternal and newborn clinics in Koboko District, Uganda: a qualitative inquiry of providers' perspective	To assess barriers and facilitators of the integrated viral hepatitis B, C and HIV care model to optimise screening uptake among mothers and newborns at health facilities in Koboko District, West Nile sub-region, Uganda.	An exploratory qualitative descriptive study was used at a Health Centre III level. Study participants were clinical and administrative health workers in Hepatitis B, C, and HIV services delivery. The data was audio and then transcribed. Data was then analysed using thematic analysis.	Koboko District, West Nile sub-region, Uganda.	Facilitators of integration were: high burden of HBV infection, health workers' team spirit, reduced long waiting time, availability of medical products, HBV and HIV integration into HMIS2 data collection tools and support availability from implementing partners. Barriers included: Knowledge gaps among HCWs, limited Health education, Language barriers, stockout of HBV test kits, and inadequate staffing.
Toskin *et al*. 2020	LMICs	Call to action for health systems integration of point-of-care testing to mitigate the transmission and burden of sexually transmitted infections	To paint a picture of the state of technology of the STI point-of-care testing (POCT) and its implications for health system integration	Material for STI POCT landscape was gathered from publicly available information, published and unpublished reports and prospectuses, and interviews with developers and manufacturers.	Publicly available information, published and unpublished reports and prospectuses, and interviews with developers and manufacturers.	There is insufficient attention to health systems capacity and conditions to ensure the swift and rapid integration of current and future STI POCT. Unless the complexity of health systems, including context, institutions, adoption systems and problem perception, is recognised and mapped, simplistic approaches to policy design and programme implementation will result in poor realisation of intended outcomes and impact.

**Table 2.1:** summary of findings for studies reporting an integrated approach to HIV, syphilis and hepatitis B management in sub-Saharan Africa

Author, Publication Year	Country	Study title	Study aim	The study methodology	Sample characteristics	Summary of Findings
Mullick *et al*. 2023	South Africa	Comparing the Integration of Syndromic versus Etiological Management of Sexually Transmitted Infections Into HIV. Pre-Exposure Prophylaxis Services for Adolescent Girls and Young Women, in SA	To describe the implementation of a syndromic compared to an etiological approach to STI integration within PrEP services in South Africa	Program data from eight fixed and four mobile clinics were analysed to describe a cascade of STI care and integration of syndromic management among clients accessing PrEP services.	22,505 clients who sought services with a high proportion (92.9%) screened for STI symptoms.	Syndromic STI screening and management can be integrated into routine PrEP service delivery and can identify symptomatic STIs, but misses asymptomatic infections. PrEP clients have a high prevalence of treatable STIs. Etiologic approaches can identify more infections than syndromic screening, but cheap point-of-care tests are needed.
Cohn *et al*. 2021	SSA	EMTCT of HIV, syphilis and HBV in SSA	To show that health systems working with communities in SSA could deliver rapid and sustainable results towards the EMTCT of all three diseases (HIV, Syphilis and HBV).	Analysis of available data and findings.	Not specified	Triple elimination is strategic to tackle HIV, syphilis and HBV through an integrated, person-centred approach. National programmes in SSA can accelerate the adoption of WHO policies and frameworks that serve to break down disease-specific vertical programmes, improve efficiency and support a person-centred and sustainable approach to triple elimination. Innovations may facilitate triple elimination programmes. Political support and the use of existing funding can realize benefits of triple elimination.
Newman *et al*. 2015	CAR, Ghana, Madagascar, Mozambique, Tanzania, and Zambia	EMTCT of HIV and syphilis: A dual approach in the African Region to improve quality of ANC and integrated disease control	To assist countries in identifying efforts towards dual EMTCT and identifying how their experiences could inform the development of a regional framework for EMTCT of HIV and syphilis	A workshop was co-convened for six SSA countries. Responses from pre-workshop questionnaires administered to country representatives and their presentations during the workshop were summarised in a Table.	Not specified	Countries in the African Region are interested in and committed to the EMTCT of HIV and syphilis in an integrated manner. Lack of service integration was noted to cause delays in dual elimination. To ensure HIV and syphilis screening and treatment services are available at a common point of service delivery. Countries like Madagascar: Activities to integrate syphilis screening into existing PMTCT health services, and Zambia: Undertaking research in dual test kits.
Phili *et al*. 2015	South Africa	Experiences in the implementation of PICT and linkage to HIV services at urban public sector health facilities in KwaZulu-Natal	To determine the feasibility and early outcomes of using the PICT strategy in KwaZulu-Natal.	This was a longitudinal, field intervention study. Health workers were trained to provide PICT to patients of two public health institutions in KwaZulu-Natal. Data were recorded and analysed using univariate and multivariate analysis.	A total of 10 806 clients were registered, with half of them from an urban hospital.	PICT integration with other services faces human resource, infrastructural and conceptual barriers, and is not currently feasible at OPDs. Operational guidance and training for providers are needed. More training and technical assistance on the implications of service integration within the existing health systems, and the improvement of data management and physical infrastructure adjustments, are required for the successful implementation of the PICT strategy. Increased on-site mentoring is needed to ensure an efficient transition from the traditional silo-based offering of services to an integrated approach to services.

**Table 2.2:** summary of findings for studies reporting an integrated approach to HIV, syphilis and hepatitis B management in sub-Saharan Africa

Author, Publication Year	Country	Study title	Study aim	The study methodology	Sample characteristics	Summary of findings
Ciccacci *et al*. 2024	Kenya	Exploring the burden of diseases in the HIV population: Results from the CHAO (Comorbidities in HIV/AIDS outpatients) cross-sectional study in Kenya	To improve understanding and management of comorbidities in HIV patients, highlighting the need for cost-effective healthcare strategies to address this dual burden.	The CHAO (Comorbidities in HIV/AIDS Outpatients) project conducted a cross-sectional epidemiological study across 25 clinics in Meru County, Kenya.	A total of 1051 HIV-positive individuals were included in the study: 75 % females, 25 % males.	There were significant comorbidities, including syphilis (5.23 %) and HBV (2.19 %). The prevalence of comorbidities varied with age. The high prevalence of comorbidities among HIV-positive patients in Meru County underscores the need for integrated healthcare strategies that address both infectious diseases and NCDs. The findings advocate for systematic screening and management of comorbidities within HIV care programs, emphasising the need for holistic health approaches to improve outcomes for this population.
Young *et al*. 2019	Kenya	Integrated POCT for HIV, syphilis, malaria and anaemia at antenatal facilities in western Kenya: a qualitative study exploring end-users’ perspectives of appropriateness, acceptability and feasibility	To capture end-users’ experiences of using POCTs for HIV, syphilis, malaria and anaemia to assess the appropriateness, acceptability and feasibility of integrated testing for ANC.	Seven dispensaries were selected to implement integrated POC testing in western Kenya. Semi-structured interviews were conducted with 18 healthcare workers trained and experienced in integrated POC testing.	18 healthcare workers from seven dispensaries in western Kenya and 118 pregnant women in 12 FGDs	Healthcare workers reported enjoying gaining new skills, were enthusiastic about using POCTs, and found them easy to use and appropriate to their practice. Initial concerns that performing additional testing would increase their workload in an already strained environment were resolved with experience and proficiency with the testing procedures. Health system challenges such as high client-to-healthcare worker volume ratio, stock-outs and poor working conditions challenged the delivery of adequate counselling and management of the four conditions. Pregnant women appreciated POCTs, but reported poor healthcare worker attitudes, drug stock-outs, and fear of HIV disclosure to their partners as shortcomings to their ANC experience in general. This study provides insights into the acceptability, appropriateness, and feasibility of integrating POCTs into ANC services among end-users. While the innovation was desired and perceived as beneficial, future scale-up efforts would need to address health system weaknesses if integrated testing and subsequent effective management of the four conditions are to be achieved.
Ejalu *et al*. 2021	Uganda	Integrating hepatitis B care and treatment with existing HIV services is possible: cost of integrated HIV and hepatitis B treatment in a low-resource setting: a cross-sectional hospital-based cost-minimisation assessment	To estimate provider costs associated with running an integrated HBV and HIV clinical pathway for patients on lifelong treatment in a low-resource setting in Uganda.	A cross-sectional cost minimisation study at two study sites that were operating separate clinics for HBV and HIV-positive clients was conducted. The project piloted the integration of the Hepatitis B clinics into the HIV ART clinics. The analysis compared the costs of running the stand-alone clinics for Arua RRH and for Koboko DH, and the integrated clinics for Arua RRH and for Koboko DH.	Data were extracted from 3121 files of HIV and HBV monoinfected patients in the West Nile region of Uganda	The application of the integrated Pathway in HIV and HBV patient management could improve hospital cost efficiency compared with operating stand-alone clinics. Findings showed that Arua Hospital had a higher cost per patient in both clinics than did Koboko Hospital. The cost per HBV patient was US$163.59 in Arua and US$145.76 in Koboko, while the cost per HIV patient was US$176.52 in Arua and US$173.23 in Koboko. The integration resulted in a total saving of US$36.73 per patient per year in Arua RRH and US$17.5 in Koboko DH.

**Table 2.3:** summary of findings for studies reporting an integrated approach to HIV, syphilis and hepatitis B management in sub-Saharan Africa

Author, Publication Year	Country	Study title	Study aim	The study methodology	Sample characteristics	Summary of findings
Young *et al*. 2019	Kenya	Integrating HIV, syphilis, malaria and anaemia POCT for ANC at dispensaries in western Kenya: discrete-event simulation modelling of operational impact	To understand the effect of providing four POC tests for ANC on nurse utilisation and wait times for women seeking maternal and child health (MCH) services, using discrete-event simulation (DES) modelling.	Time-motion data were collected from one dispensary in Kenya. A simulation model was constructed. The intervention from the model was removed to obtain wait times, length-of-stay and nurse utilisation rates for the baseline scenario where only HIV testing was offered for ANC.	A total of 183 women visited the dispensary for MCH services, and 14 of these women received POC testing	The model suggests there was sufficient time to deliver all the WHO’s required ANC activities and offer integrated testing for ANC first and re-visits with the current number of healthcare staff. Further investigations on improving healthcare worker availability, performance and quality of care are needed. Delivering four POC tests together for ANC at the dispensary level would be a low-burden strategy to improve ANC.
Manabe *et al*. 2015	Uganda	Integration of antenatal syphilis screening in an urban HIV clinic: a feasibility study	To investigate the feasibility of adding syphilis screening within an integrated antenatal HIV clinic.	A descriptive cohort study was conducted of HIV-infected pregnant women at the Infectious Diseases Institute (IDI) in Kampala, Uganda. Enrolled women were tested using rapid plasmin reagin. If positive, they were treated.	A total of 584 of 606 (95.7%) women approached and consented to test for syphilis.	Structural interventions such as opt-out testing for syphilis within integrated HIV-antenatal care clinics are feasible and capitalise on the excellent care programs that have already been established for HIV care. Novel approaches are required for partner notification.
Baseke *et al*. 2015	Uganda	Prevalence of hepatitis B and C and relationship to liver damage in HIV infected patients attending Joint Clinical Research Centre Clinic (JCRC), Kampala, Uganda.	To determine the prevalence of HBV, HCV, their co-infection with HIV and their effect on liver cell function.	This was a cross-sectional study conducted at the Joint Clinical Research Centre (JCRC) among HIV positive individuals attending the clinic after consenting. Serum was collected for the detection of HBsAg, HCV-specific antibodies and ALT liver enzyme.	A total of 89 patients were enrolled.	HBV prevalence was high in HIV positive individuals, with more women commonly infected. This study revealed a high prevalence of liver cell injury among HIV positive individuals, although the injury due to HBV or HCV infection was lower than that which has been documented. From this study, the high prevalence of HBV and HCV among HIV positive individuals pointed to a need for screening of HIV positive individuals for the hepatitis viruses.
Peeling *et al*. 2017	Not Specified	The future of viral hepatitis testing: innovations in testing technologies and approaches.	To examine a range of other technological innovations that can be leveraged to provide more affordable and simplified approaches to testing for HBV and HCV infection and monitoring of treatment response.	An analysis was done on improved access to testing through health system improvements, such as integration of laboratory services for procurement and sample transportation and enhanced data connectivity to support quality assurance and supply chain management.	Not specified	Viral hepatitis services and particularly hepatitis B management in SSA may also be integrated into the existing infrastructure of HIV programmes for testing, care and treatment. Innovations in testing and sampling approaches have the potential to increase access to testing and reduce the large burden of undiagnosed infection.

**Table 2.4:** summary of findings for studies reporting an integrated approach to HIV, syphilis and hepatitis B management in sub-Saharan Africa

Author, Publication Year	Country	Study title	Study aim	The study methodology	Sample characteristics	Summary of findings
Tucker *et al*. 2017	Global including Nigeria	The HepTest Contest: a global innovation contest to identify approaches to hepatitis B and C testing	To identify examples of different hepatitis B and C approaches to support countries in their scale-up of hepatitis testing, and to supplement the development of formal recommendations on service delivery in the 2017 World Health Organization Hepatitis B and C testing guidelines.	This contest involved four steps: 1) establishment of a steering committee to coordinate a call for contest entries; 2) dissemination of the call for entries through (Facebook, Twitter, YouTube, email listservs, academic journals); 3) independent ranking of submissions according to prespecified criteria (clarity of testing model, innovation, effectiveness, next steps) using a 1-10 scale; 4) recognition of highly ranked entries through presentation at international conferences, commendation certificate, and inclusion as a case study in the WHO 2017 testing guidelines	A total of 27 countries, including Nigeria	A variety of different testing delivery approaches were employed, including integrated HIV-hepatitis testing (n = 12); integrated testing with harm reduction and addiction services (n = 9); use of electronic medical records to support targeted testing (n = 8); decentralisation (n = 8); and task shifting (n = 7). The global innovation contest identified a range of local hepatitis testing approaches that can be used to inform the development of testing strategies in different settings and populations. Further implementation and evaluation of different testing approaches are needed.
Balira *et al*. 2015	Tanzania	The need for further integration of services to prevent mother-to-child transmission of HIV and syphilis in Mwanza City, Tanzania	To assess the operational integration of maternal HIV testing and syphilis screening in Mwanza, Tanzania.	Interviews were conducted with 76 health workers (HW) from three antenatal clinics (ANC) and three maternity wards in 2008–2009, and 1137 consecutive women admitted for delivery. Nine ANC health education sessions and client flow observations were observed.	A total of 76 health workers (HW) from three antenatal clinics (ANC) and three maternity wards in and 1137 consecutive women were admitted for delivery.	Only 25.0% of HWs reported having received training in PMTCT and syphilis screening. HIV and syphilis tests were sometimes performed in different rooms and results recorded in separate registers with different formats, and the results were not always given by the same person. Integration of maternal HIV and syphilis screening was limited. Integrated care guidelines and related health worker training should address this gap.
Hutin *et al*. 2017	Not Specified	Viral Hepatitis Strategic Information to Achieve Elimination by 2030: Key Elements for HIV Program Managers	To present a viewpoint on Viral Hepatitis Strategic Information to Achieve Elimination by 2030: Key Elements for HIV Program Managers	Information was gathered from worldwide sources to prepare a viewpoint.	Not Specified	The many commonalities between HIV and HBV in terms of the diseases that they cause and the responses required justify the implementation of integration. This should be considered at strategic, policy, technical, and implementation levels.

**Table 3:** thematic analysis summary of HSs' integrated approach systematic review studies for triple elimination in SSA

Main theme	Sub-theme	Studies
Effectiveness improved by HSs' integration	Services delivery effectiveness	Newman *et al*. 2015 and Ejalu *et al*. 2021
	Health systems financing effectiveness	
HSs Barriers to integration	Human resources barriers	Alege *et al*. 2025; Toskin *et al*. 2020; Mullick *et al*. 2023; Phili *et al*. 2015; Young *et al*. 2019; Balira *et al*. 2015
	Services delivery barriers	
	Medical devices barriers	
	Health systems financing barriers	
	Health information management barriers	
	Structural barriers	
HSs Facilitators of the integrated approach to triple elimination	Services delivery facilitators	Thompson *et al*. 2021; Alege *et al*. 2025; Toskin *et al*. 2020; Mullick *et al*. 2023; Cohn *et al*. 2021; Newman *et al*. 2015; Ciccacci *et al*. 2024; Young *et al*. 2019; Manabe *et al*. 2015; Hutin *et al*. 2017
	Leadership and governance facilitators	
	Health systems financing facilitators	
	Human resources for health facilitators	
	Medical devices facilitators	
	Health information management facilitators	
	Structural facilitators	
Improved case detection	More HBV cases detected among HIV positives	Baseke *et al*. 2015; Peeling *et al*. 2017, and Balira *et al*. 2015
	More syphilis testing access when offered together with HIV testing services	
Recommendation for HSs integration	Human resources for health recommendations	Mathebula, *et al*. 2020; Thompson *et al*. 2021; Cohn *et al*. 2021; Newman *et al*. 2015; Phili *et al*. 2015; Ciccacci *et al*. 2024; Young *et al*. 2019; Peeling *et al*. 2017; Tucker *et al*. 2017; Balira *et al*. 2015; Hutin *et al*. 2017
	Health services delivery recommendations	
	Medical devices recommendations	
	Leadership and governance recommendations	
	Health systems financing recommendations	
	Health information management systems recommendations	
	Structural recommendations	

**Thematic analysis of included studies:** five themes were developed: a) effectiveness of HSs integration, b) barriers to HSs integration, c) facilitators for HSs integrated approach on triple elimination, d) improved case detection, and e) recommendations for HSs integration. All themes except the one on improved case detection were developed from sub-themes built generally around six pillars of the health systems according to WHO, namely Human Resources for Health, Health Services Delivery, Medical Devices, Leadership and Governance, Health Systems Financing and Health Information Management Systems.

***Effectiveness of HSs integration:*** it was noted that the lack of integration had negative effectiveness as it would delay EMTCT of syphilis and HIV [26]. Evidence showed that HIV and HBV patient management through an integrated approach could improve hospital cost efficiency when compared with operating stand-alone clinics [27].

***Barriers to HSs integration:*** a number of barriers to HSs integrated approach to services that included HIV, syphilis and HBV were highlighted. These included gaps in knowledge among healthcare workers, inadequate health education, difficulties in communication between mothers and health workers due to language barriers, persistent HBV test kits stockout, inadequate staffing [28], lack of independent laboratory test kits evaluation, including at points of care, and lack of accurate, rapid and affordable POCTs. There is insufficient attention being paid to health systems' capacity and conditions for integration of the services [29]. There are insufficient cheap point-of-care tests leading to syndromic screening [30]. Human resources, conceptual and infrastructural barriers exist against HSs integration [31]. Human resources are concerned that their workload would increase due to performing additional testing under an already strained environment, general health system challenges that included a high volume ratio of client to healthcare worker, and poor working conditions were some of the barriers [32]. It was noted from the same study that poor attitudes from healthcare workers and fear of HIV disclosure by clients to their partners were some of the shortcomings. A low (25.0%) number of HWs had received training in PMTCT of HIV and syphilis, including screening in some settings [33]. The same study noted that, sometimes, HIV and syphilis tests were performed in different rooms with separate registers used to record results using different formats, while results were not given by the same person all the time.

***Facilitators for HSs integrated approach to triple elimination:*** HBV infection birth-dose vaccination, integrated within the current HIV PMTCT programme, catalyses HBV elimination progress in sub-Saharan Africa (SSA) [34]. Recognising and mapping health systems complexity, including context, institutions, adoption systems and problem perception, facilitates the integrated approach [29]. The high burden of MTCT in SSA for the three infections (HIV, syphilis and HBV), would benefit from HSs integrated approach as an adopted strategy to promote triple elimination [7]. Political support that can be secured by SSA health community for the use of existing funding facilitates HSs integrated approach to realise the benefits of triple elimination because countries in the African Region are interested and committed to triple EMTCT in an integrated manner. They also recognise that EMTCT using an integrated approach is feasible through strengthening the maternal, newborn and child health platform [26]. High prevalence of HIV and its comorbidities in the region underscores the need for integrated healthcare strategies [35]. The enthusiasm among health care workers who reported enjoying gaining new skills using POCTs that they found easy to use and appropriate to their practice facilitates HSs integrated approach. The availability of diagnostic tools and sufficient delivery time of WHO's required ANC integrated activities for first and re-visits facilitates the approach with the current number of HCWs in some settings [32]. Structural interventions within integrated HIV-ANC clinics facilitate HSs integrated approach [36]. There are many commonalities between HIV, HBV, and other comorbidities, which facilitate HSs integrated approach. These include diseases they cause, responses required that facilitate coordination, strategic linkages or integration of programs according to the respective magnitudes of the epidemics. Similarities in the SDG targets for HIV and some comorbidities facilitate the overarching priority to integrate other diseases and HIV strategic information within single broader existing health information systems. This contributes toward strengthening the health systems [37].

***Improved case detection:*** integrated screening for HIV and HBV demonstrated a high prevalence of HBV among HIV-positive individuals, with more women commonly infected, pointing to the importance of an integrated approach for improved case finding [38]. Innovations in integrated testing and sampling approaches have the potential to increase access to testing and reduce the large burden of undiagnosed infection [39]. Syphilis diagnosis and management increase when integrated with HIV services [33].

***Recommendations for HSs integration:*** improved integrated HIV and STI prevention and management is needed [40]. Both HBV screening and treatment of women in pregnancy need to be added to existing HIV PMTCT platforms because it is feasible [34]. National programmes among countries in SSA can increase adoption of WHO policies and frameworks meant to break down disease-specific vertical programmes, promote efficiency and improve a person-centred, sustainable triple elimination approach. Innovations (like HSs integrated approach) are recommended to facilitate triple elimination programmes [7]. HIV and syphilis screening and treatment services are encouraged to be available at common service delivery points. Activities are recommended to integrate syphilis screening into existing PMTCT health services through research on test kits. Experiences in SSA countries are recommended to assist regional and global efforts for dual EMTCT of HIV and syphilis in addition to strengthening ANC services [26]. There is a need for urgent provision of operational guidance and training for health care providers. More training and technical assistance on the implications of service integration within existing health systems, and the improvement of data management and physical infrastructure adjustments are required to ensure the successful implementation. Incremental investment in the data system that is coordinated is required to provide the evidence base needed for guiding the elimination of viral hepatitis and comorbidities. Increased on-site mentoring is recommended to ensure an efficient transition to an integrated approach to services from the traditional silo-based offering of services [31]. The findings advocate for systematic screening and management of comorbidities within HIV care programs, emphasising the need for holistic health approaches to improve outcomes [35]. If integrated testing and subsequent best management of HIV, syphilis and HBV are to be achieved, future scale-up efforts have to address health system weaknesses [32]. Further investigations are needed on improving healthcare worker availability, performance and quality of care in integrated ANC services. Delivering point-of-care tests in an integrated manner would be a low-burden strategy at the dispensary level to improve services at ANC [41]. Viral hepatitis services in SSA need to be integrated into the existing HIV programmes infrastructure for testing, care and treatment [39]. Implementation and further evaluation of different testing approaches are required [42]. Similarly, integrated approach guidelines to maternal HIV and syphilis screening, which are limited, need to address human resources gaps related to training [33]. It is recommended that health systems articulations have to be considered at the strategic, policy, technical, and implementation levels [37].

## Discussion

Triple elimination framework seeks to integrate HIV, syphilis and viral hepatitis programmes by better service delivery coordination among programmes through incorporation of HBV screening into existing HIV and syphilis screening at ANC [43]. No recent studies have been identified to explore HSs barriers and facilitators in the integration of HIV, syphilis, and HBV programs into ANC services for triple elimination since its implementation in 2018 in other settings [44]. In sub-Saharan Africa (SSA), high burden of the diseases, poor population health outcomes and inequities are due to weak health systems [45]. This makes it difficult to adopt an HSs integrated approach to triple elimination. However, integrating HSs to monitor the three diseases targeted for elimination is needed [46].

In this systematic review, we identified 18 studies documenting the integration of HIV and syphilis, HIV and hepatitis or HIV and any other services. In the findings, none of the reviewed studies reported integration of all three diseases in a comprehensive, triple elimination framework. None reported integration in the context of clearly coordinated, envisioned WHO HSs pillars. These findings highlight significant gaps in the literature and practice, as well as a salient disconnection between global policy recommendations and the current state of implementation in sub-Saharan Africa. The findings suggest that, while some progress has been made towards dual integration, the full realisation of an integrated approach targeting all three infections simultaneously has not been achieved in sub-Saharan Africa.

The other major finding was the identification of five key themes currently summarising the landscape of HSs integration efforts towards triple elimination in sub-Saharan Africa. Thematic analysis revealed a potential for integrated approaches to enhance the effectiveness of health systems, leading to improved HIV, syphilis and HBV service delivery and health outcomes. For the effectiveness to be achieved, significant barriers within the WHO's HSs pillars can work against the full potential of integrated care. On the other hand, several facilitators that can be grouped within WHO's HSs pillars can work to the full potential of integrated care as identified, to drive progress towards the triple elimination goals. Integration was associated with improved case detection, particularly in ANC settings, reinforcing the value of combining services towards triple elimination. Recommendations covering all the WHO HSs pillars for advancing an integrated approach were highlighted, emphasising context-specific strategies promoting progress towards triple elimination in line with global health sector strategies.

Findings from this study agree with prior systematic reviews and studies, which similarly noted that while dual integration of HIV and syphilis, or HIV and hepatitis, is feasible and beneficial, triple integration of all three diseases has not been adequately studied in SSA [47,48]. Our findings build on previous findings, which noted that several health system factors either facilitate or act as barriers to integrated service provision for triple elimination of vertical transmission [49]. Important implications for health policy and practice in sub-Saharan Africa emerged from findings of this review. The sub-themes that emerged demonstrate the need to consider barriers and facilitators across health systems pillars when implementing the integrated approach to triple elimination. Despite the WHO strongly advocating for a triple elimination framework and providing clear guidance on integrating services across its six HSs pillars, a notable lack of studies explicitly evaluating or implementing integration in alignment with these pillars exists. Lack of studies specific to triple integration suggests an urgent need for pilot projects and operational research to test and refine models that can deliver such comprehensive services for HIV, syphilis, and hepatitis in an integrated manner. Literature shows that there is a need for more evidence on integration in specific geographical areas, such as SSA, because the success of integration strategies is highly dependent on context [50]. Addressing the identified barriers, while promoting the facilitators, will be essential for scaling up integrated approaches, accelerating progress towards targets for triple elimination set by the WHO.

## Conclusion

While there is evidence of progress in integrating HIV and syphilis, HIV and HBV as well as HIV and other services, a fully integrated HSs approach for triple elimination has not been fully implemented in sub-Saharan Africa (SSA). The thematic analysis highlights both the promise and the challenges of integration, offering a roadmap for future efforts to strengthen HSs and achieve public health goals in the region. Further enquiry on the use of HSs integrated approach needs to be considered for realization of triple elimination.

### What is known about this topic

- Health systems integrated approaches are increasing in developed countries such as the United States, United Kingdom and Australia and are increasingly recognized as critical for addressing multiple public health threats, including HIV, syphilis, and viral hepatitis, in sub-Saharan Africa;
- Although progress exists towards elimination targets of HIV, syphilis, and viral hepatitis in sub-Saharan Africa, significant barriers remain, such as vertical programming and limited routine screening, which hinder sustainable progress in eliminating these infections as public health threats;
- There is no identified dominant health system integration theory, model or framework, suggesting that a one-size-fits-all solution may not be effective.

### What this study adds

- This systematic review identifies, retrieves and provides a comprehensive synthesis of international evidence on utilization of HSs integrated approach on the elimination of HIV, syphilis and viral hepatitis as public health threats in SSA;
- The study provides actionable recommendations for practice, policymakers and health system managers, emphasizing strategies to strengthen integration, improve service delivery, and accelerate progress towards elimination goals in the region, including further research;
- Key insights on the effectiveness, facilitators, and barriers to full integration of services for triple elimination within the context of WHO's health systems 6 pillars.

## Annex

**Annex 1**: supplementary material (PDF 250KB)
